# Case of Aneurism of the External Iliac, in Which a Ligature Was Applied to the Common Iliac Artery

**Published:** 1844-09-21

**Authors:** Richard Hey

**Affiliations:** Surgeon to the York County Hospital


*Case of Aneurism of the External Iliac, in which a Ligature was applied to the Common Iliac Artery.* By Richard Hey, Esq., F.R.C.S., Surgeon to the York County Hospital.

The patient, a gentleman aged 40, perceived, on the 10th of November, a small tumour in his left groin, above the centre of Poupart's ligament. Three days afterwards he had severe pain in the part, and on the following day the swelling increased in size, accompanied with pulsation. The tumour gradually enlarged, assumed at one part a conical shape, and the skin was tense and red. By moderate continued pressure the swelling could be reduced in size, but it resumed its former magnitude when the pressure was taken off. It was resolved, in consultation, to apply a ligature to the common iliac artery, which operation was performed by the author on December 3d. At this time the tumour measured six inches across, in a transverse direction, and projected three inches from the plane of the abdomen. At one point it was about an inch and a half from the navel. The incision was begun two inches and three quarters above the navel, and three inches from the median line, and was carried six inches downwards, in a semicircular direction, with a prolongation of an inch and a half, in a straight line, outwardly. The layers of muscles and fascia transversalis having been divided to the whole extent of the incision, the peritoneum was gently separated from the parts beneath, and the common iliac artery was easily reached. A little time was occupied in scratching through the sheath with the point of the aneurism needle, after which it was passed under the artery, from within outwards, armed with a double ligature of staymaker's silk, and the operation completed. The pulsation in the tumour ceased immediately after the artery was tied. The patient proceeded, on the whole, favourably after the operation, except that he was in great danger at one time, from an accumulation of hardened fæces in the rectum. The tumour gradually subsided in size, and both legs were nearly of the same temperature, the affected limb being surrounded with flannel. A week after the operation, pulsation was felt in the anterior tibial artery. On the twenty-eighth day the ligature was found loose in the wound and removed. About the twentieth of January the patient was free from complaint, and was able to walk about.

Sir G. Lefevre inquired into the statistics of the operation in question, and referred to one which had been performed successfully by Dr. Salaman, a Russian surgeon, and which he, (the speaker,) had some years since transmitted to the Society. It had afterwards been published in the Medical Gazette. In this case, the most troublesome symptom after the operation was the occurrence of sphacelus of the integuments of the knee and ankle on the affected side.

The President referred to the case of Mr. Guthrie, in which the common iliac was tied, for a tumour which was supposed to be aneurismal, but was found afterwards to be funguoid. Sir Philip Crampton had performed the operation; the case terminated fatally, by the occurrence of secondary hæmorrhage. Dr. Mott's case was successful.

Mr. Hey referred to an article in the first volume of the Medical Gazette for 1842–3, in which all the operations, whether successful or otherwise, were mentioned. Among them was the case of Dr. Salaman. Sir Philip Crampton had attributed the failure of his operation to his having employed a cat-gut ligature, which he supposed had become absorbed, and had given rise to fatal hæmorrhage. Mr. Liston had tied the common iliac for secondary hæmorrhage, but without success. Of the cases mentioned in the article referred to, only three or four were successful.—*Trans. Royal Medical and Chirur. Society of Lon., from Lon. & Ed. Mon. Jour. of Med. Science.*

